# A Sensitive Reverse Transcription Loop-Mediated Isothermal Amplification Assay for Direct Visual Detection of SARS-CoV-2

**DOI:** 10.4269/ajtmh.20-1079

**Published:** 2020-10-23

**Authors:** Yee Ling Lau, Ilyiana Binti Ismail, Nur Izati Binti Mustapa, Meng Yee Lai, Tuan Suhaila Tuan Soh, Afifah Haji Hassan, Kalaiarasu M. Peariasamy, Yee Leng Lee, Pik Pin Goh

**Affiliations:** 1 Department of Parasitology, Faculty of Medicine, University of Malaya, Kuala Lumpur, Malaysia;; 2 Department of Pathology, Hospital Sungai Buloh, Selangor, Malaysia;; 3 Clinical Research Centre, Hospital Sungai Buloh, Selangor, Malaysia;; 4 Institute for Clinical Research (ICR), National Institutes of Health (NIH), Ministry of Health Malaysia, Putrajaya, Malaysia

## Abstract

A simple and rapid reverse transcription loop-mediated isothermal amplification (RT-LAMP) assay was developed for the detection of SARS-CoV-2. The RT-LAMP assay was highly specific for SARS-CoV-2 and was able to detect one copy of transcribed SARS-CoV-2 RNA within 24 minutes. Assay validation performed using 50 positive and 32 negative clinical samples showed 100% sensitivity and specificity. The RT-LAMP would be valuable for clinical diagnosis and epidemiological surveillance of SARS-CoV-2 infection in resource-limited areas as it does not require the use of sophisticated and costly equipment.

The rapid spread of COVID-19 represents a global public health threat with a significant impact on the global economy, health, and many other sectors. COVID-19 is caused by coronavirus called SARS-CoV-2.^1^ Quantitative reverse transcription PCR (RT-qPCR)^2^ assays have been developed for the rapid detection of SARS-CoV-2, but they often require costly and sophisticated PCR machines which hamper its application in resource-limited settings.

Loop-mediated isothermal amplification (LAMP) represents a potential alternative for molecular testing in these settings. Loop-mediated isothermal amplification can amplify nucleic acids under isothermal conditions using a simple incubator or water bath with high speed, specificity, and sensitivity. Recently, this assay has been successfully used to detect pathogenic viruses such as Newcastle disease virus, classical swine fever virus, and SARS-CoV-2.^3–5^ Loop-mediated isothermal amplification is highly specific because of the use of four specific primers (Forward primer [F3], Backward primer [B3], Forward inner primer [FIP], and Backward inner primer [BIP]) that recognize six independent regions of a target DNA fragment. Forward inner primer and BIP bind to four distinct regions of the target fragment. The duration of LAMP reaction can be shortened by the addition of loop primers (loop forward [LF] and loop backward [LB]) and swarm primers (Forward swarm primer [F1S] and Backward swarm primer [B1S]).^6^ Loop primers targeted between the primering sites of FIP/BIP primers and swarm primers targeted on a region upstream of the FIP/BIP primers recognition sequences on opposite strands (Martineau et al., 2017). This modification has shown to reduce the reaction time and at the same time, increase sensitivity. The primer sequences are shown in Table 1. The reverse transcription LAMP (RT-LAMP) assay was performed in a 12.5-μL reaction mixture that consisted of 4.65 µL RNase-free water, 1X isothermal amplification buffer, 6 mM MgSO_4_, 1.2 mM deoxyribonucleotide triphosphates 4 U *Bacillus stearothermophilus* 2.0 WarmStart DNA polymerase, 3 U WarmStart RTx reverse transcriptase (NEB, Ipswich, MA), 0.5 µL RNasin^®^ Ribonuclease Inhibitor (Promega, Madison, WI), 6 µM FIP and BIP primers each, 0.4 µM LF and LB primers each, 0.2 µM F3 and B3 primers each, 1.6 µM F1S and B1S swarm primers each, and 2 µL of RNA template. The reaction mixture was incubated at 65°C in a Loopamp Real-Time Turbidimeter LA 500 (Eiken Chemical Co., Ltd., Taito-ku, Japan). In the present study, a previously developed RT-LAMP^5^ was optimized to detect SARS-CoV-2 in clinical samples within 24 minutes by additional of swarm primers. The best performance of RT-LAMP reaction was achieved with the eight primer sets (FIP/BIP, F1S/B1S, F3/B3, and LF/LB) with 1.6 µM of each swarm primer concentration. The optimal LAMP reaction was observed at 65°C. Detection of LAMP amplification products was performed by observing the color changes of the reaction tube after the additional of hydroxy naphthol blue (HNB)^7^ (Figure 1), which reduces the cost and labor of post-amplification analysis.

**Table 1:** Primers used in this study

Primer	Sequence (5′–3′)
Forward inner primer	TGGGGTCCATTATCAGACATTTTAGTTTTAGAGTATCATGACGTTCG
Backward inner primer	CGAAATGCACCCCGCATTACCCACTGCGTTCTCCATTC
Forward loop primer	TGTTCGTTTAGATGAAATC
Backward loop primer	TGGTGGACCCTCAGATTCAA
Forward primer	GTTGTTCGTTCTATGAAGACT
Backward primer	GACGTTGTTTTGATCGCG
Forward swarm primer	TTGGGGTCCATTATCAG
Backward swarm primer	ATCAGCGAAATGCACC

**Figure 1.**
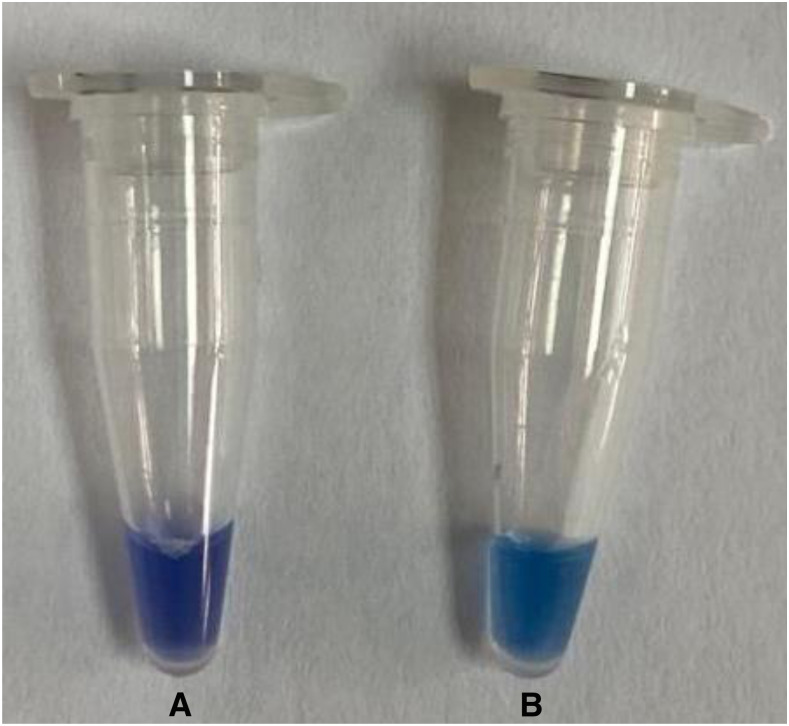
Detection of loop-mediated isothermal amplification (LAMP) products by the color change method using hydroxy naphthol blue. (**A**) The negative LAMP reaction remained in violet color (**B**), whereas the positive reaction was indicated by a color change from violet to sky blue.

The RT-LAMP was highly specific for detecting SARS-CoV-2. The results showed that the amplification product was positive only when SARS-CoV-2 RNA was added. The RNA of other virus strains could not be detected. The specificity of the RT-LAMP assay was determined by using genomic RNA of adenovirus, human metapneumovirus, influenza A (A/H1pdm2009 and A/H3) viruses, influenza B virus, parainfluenza virus 3, rhinovirus A, respiratory syncytial virus B, and enterovirus D68. Results indicate that none of these virus strains was positive.^5^ The 10-fold serial diluents of SARS-CoV-2 in vitro–transcribed RNAs were used as a template for both RT-LAMP and RT-qPCR amplification. The detection limit of RT-LAMP was one copy and that of RT-qPCR was five copies. When tested with 50 RT-PCR–positive (cycle threshold levels between 21 and 38 cycles) and 32 RT-PCR–negative nasopharyngeal swabs samples collected from a recent COVID-19 outbreak in Malaysia by Hospital Sungai Buloh, Malaysia, RT-LAMP showed a sensitivity and specificity of 100%, with an average incubation time of 24 minutes.

Reverse transcription loop-mediated isothermal amplification assay has been widely developed and used in nucleic acid amplification as it is rapid, simple, sensitive, and specific. Recently, this assay has been successfully used for the diagnosis of pathogenic microorganisms and in drug resistant studies.^8,9^ To overcome the limitations of RT-qPCR which is costly, we have optimized a previously developed RT-LAMP assay^5^ by supplementation of swarm primers for the rapid detection of SARS-CoV-2. The swarm primers prime at the F1/B1 sites of the FIP/BIP primer recognition sequences and further enhance the amplification process without complications.^6^ Following the adding of this primer set, we optimized the combination of primer sets, concentration of swarm primers, and temperature to reduce the detection time. The LAMP reaction was optimized at 65°C using all eight primers together with 1.6 µM of each swarm primer. Reverse transcription loop-mediated isothermal amplification with swarm primers improved average reaction time from 30 to 24 minutes. Meanwhile, the sensitivity of the RT-LAMP assay was evaluated through tenfold serial dilution of transcribed RNA which showed a detection limit of one copy per reaction, supporting the higher sensitivity of this assay than RT-qPCR (five copies) and other RT-LAMP assays developed by Lu et al.^10^ and Baek et al.,^11^ which has a detection limit of 118.6 and 100 copies per reaction, respectively. When tested with clinical samples, the RT-LAMP assay developed in this study showed a sensitivity and specificity of 100%. The results promote its application in detecting a low viral load infection. The LAMP approach has been combined with the recombinase polymerase amplification by El-Tholotha et al.^12^ for the detection of SARS-CoV-2. This study is of greater sensitivity than LAMP alone, but the whole process needs ∼1 hour to complete. On top of that, this assay has not been tested with actual clinical samples.

Given that RT-LAMP is highly sensitive, contamination is a major problem for end point analysis, which required the reaction tube to be opened, which can generate aerosols, such as agarose gel analysis and sequencing. To overcome this problem, we used HNB as a color indicator which resulted in a color change for positive reaction. This is cost-effective and simple to use as described by Goto et al.^7^

However, the major limitation of this assay compared with RT-qPCR is the complexity of designing LAMP primers. Loop-mediated isothermal amplification requires the design of a set of at least four primers to identify six regions of the target gene to increase the efficiency of the reaction, with the inclusion of two additional swarm primers to accelerate the reaction. As such, primer development could be challenging.

In this study, we optimized a SARS-CoV-2–specific RT-LAMP assay with the inclusion of swarm primers as a rapid, simple, sensitive, and specific molecular test for the detection of SARS-CoV-2. Sensitive and rapid diagnosis assay that can recognize early infection can be very important for control of the disease in affected communities.
